# Magnetic Sphincter Augmentation Versus Fundoplication in Non-obese Gastroesophageal Reflux Disease (GERD) Patients: A Systematic Review of Patient-Reported Outcomes and Dysphagia

**DOI:** 10.7759/cureus.93599

**Published:** 2025-09-30

**Authors:** M Shazan Raza, Maryam Sadiq, Naeem Iqbal, Noman Sadiq, Muniba Zafar

**Affiliations:** 1 General Surgery, Rawalpindi Medical University, Rawalpindi, PAK; 2 Medicine and Surgery, Rawalpindi Medical University, Rawalpindi, PAK; 3 General Surgery, Bahria University Medical and Dental College, PNS (Pakistan Naval Ship) Shifa Hospital, Karachi, PAK

**Keywords:** belching, gerd, laparoscopic fundoplication, linx device, magnetic sphincter augmentation, non-obese patients, proton pump inhibitors, surgical outcomes

## Abstract

Magnetic sphincter augmentation (MSA) and laparoscopic fundoplication (LF) are established surgical treatments for gastroesophageal reflux disease (GERD). While several comparative studies exist, evidence specifically focusing on non-obese populations remains less extensively characterized. This systematic review evaluates the effectiveness, functional outcomes, and safety profiles of MSA versus LF in non-obese adults with GERD. The protocol was registered with Prospective Register of Systematic Reviews (PROSPERO: CRD420251106241). Following Preferred Reporting Items for Systematic Reviews and Meta-Analyses (PRISMA) guidelines, a comprehensive literature search was conducted across PubMed, ScienceDirect, Cochrane Library, and Google Scholar. Comparative studies published from 2015 to 2025 were included if they reported outcomes of MSA and LF in non-obese adults (BMI < 30). Data extraction focused on demographics, GERD-Health Related Quality of Life (GERD-HRQL), proton pump inhibitor (PPI) discontinuation, dysphagia, bloating, belching, and complications. Risk of bias was assessed using the Newcastle-Ottawa Scale. Eight studies with 1,598 patients met the inclusion criteria; 1,078 underwent MSA and 520 received LF. Both interventions significantly improved GERD-HRQL scores. Across the five studies reporting PPI discontinuation, rates were numerically higher with MSA in three studies and higher with LF in two. Overall, findings were mixed and no consistent advantage emerged. MSA patients reported better preservation of belching and reduced bloating. However, persistent dysphagia was more frequent in MSA, with predictors including preoperative dysphagia and weak esophageal motility. Long-term data indicated comparable GERD control but slightly higher reoperation and hernia recurrence rates with MSA. Patient satisfaction was high in both groups, with MSA favored for function-preserving outcomes such as reduced bloating and preserved ability to belch and vomit. MSA and LF both offer effective symptom relief in non-obese GERD patients. MSA demonstrates advantages in physiological function and reversibility but carries a higher risk of early dysphagia. It may be a preferred option in well-selected patients, although further randomized trials are needed to define its role as a primary surgical choice.

## Introduction and background

Gastroesophageal reflux disease (GERD) is a condition that develops when there is a retrograde flow of stomach contents back into the esophagus, primarily due to lower esophageal sphincter (LES) dysfunction. It can present as non-erosive reflux disease or erosive esophagitis [1]. Gastroesophageal reflux disease is one of the most frequent diseases encountered by primary care providers. The typical and atypical symptoms of GERD include heartburn, regurgitation, globus sensation, dysphagia, chest pain, and belching [2]. A valve mechanism exists between the esophagus and the stomach, formed by the lower esophageal sphincter (LES), the diaphragm, the His angle, the Gubaroff valve, and the phrenoesophageal membrane [3]. Physiologically, the LES is a 3- to 4-cm-long segment of tonically contracted smooth muscle at the distal end of the esophagus [4]. A functional (frequent transient LES relaxation) or mechanical (hypotensive LES) problem of LES is the primary contributor to GERD. Treatment options available for GERD include lifestyle modifications, over-the-counter and prescription medications (antacids, proton pump inhibitors (PPIs), H2 blockers), endoscopic therapies (e.g., transoral incisionless fundoplication), and surgical treatment (e.g., magnetic sphincter augmentation, laparoscopic fundoplication) [5].

Patients who present with medically refractory GERD, noncompliance or side effects with medical therapy, a large hiatal hernia, or who desire to discontinue long-term medical treatment can be considered for surgical management [6]. Among patients considered for surgery, non-obese individuals with medically refractory GERD represent a clinically important group warranting focused evaluation. Unlike obese patients who may be candidates for bariatric procedures, non-obese patients require alternative surgical options tailored specifically to reflux management. The available surgical options for GERD include laparoscopic Nissen fundoplication (total 360°), Toupet fundoplication (partial posterior 270°), Dor fundoplication (partial anterior 180°), and Watson fundoplication (anterior 180°). In patients with GERD and significant obesity (BMI ≥ 35 kg/m² with comorbidities or ≥ 40 kg/m²), however, Roux-en-Y gastric bypass is often preferred over sleeve gastrectomy as it tends to provide better reflux control [7]. Laparoscopic Nissen fundoplication has been the gold standard surgical treatment in the management of GERD patients which is a technique to recreate lower esophageal sphincter pressure by wrapping the fundus of the stomach 360° posteriorly around the esophagus in the abdomen [8]. It can successfully eliminate GERD symptoms and improve quality of life and significantly reduce the need for chronic GERD medical treatment [9].

While traditional surgical options have an acceptable safety profile, there has been an increasing interest in alternate treatments that may potentially offer similar results and be associated with a faster recovery [10]. In the light of this, many different types of endoscopic therapies have been developed for GERD management. The currently available endoluminal therapies include transoral incisionless fundoplication (TIF) using the EsophyX device (EndoGastric Solutions, Redmond, Washington, United States), radiofrequency energy delivery (Stretta procedure), and anti-reflux mucosectomy (ARMS), among others. Magnetic sphincter augmentation (MSA), on the other hand, is a minimally invasive laparoscopic surgical option. MSA is a procedure where the esophagogastric junction barrier is augmented using a bracelet of magnetized titanium beads [11]. In the closed position, the highest attractive force between the magnetic beads would reinforce the lower esophageal sphincter to strengthen the antireflux barrier while preserving normal esophageal physiology [12]. Given the increasing emphasis on quality of life and functional recovery, this systematic review aims to compare MSA and LF in terms of patient-reported outcomes, dysphagia, and related postoperative symptoms.

## Review

Materials and methods

Protocol and Reporting Guidelines

The protocol for this systematic review was registered in the International Prospective Register of Systematic Reviews (PROSPERO; Registration No: CRD420251106241). This systematic review was conducted in accordance with the Preferred Reporting Items for Systematic Reviews and Meta-Analyses (PRISMA) guidelines [13].

Eligibility Criteria

We included comparative studies that enrolled adults aged 18-65 years with gastroesophageal reflux disease (GERD) and a body mass index (BMI) of less than 30, thereby excluding obese and pediatric populations. Eligible interventions consisted of magnetic sphincter augmentation (MSA) using the LINX device (Ethicon, Cincinnati, Ohio, United States), with laparoscopic fundoplication (either Nissen or partial) as the comparator. Studies were required to report at least one relevant outcome, including GERD-Health Related Quality of Life (GERD-HRQL) [14], postoperative dysphagia, discontinuation of proton pump inhibitors (PPIs), or the ability to belch or vomit. We considered prospective and retrospective cohort studies, case-control studies, and propensity score-matched studies published in English between 2015 and 2025. Non-comparative studies, case reports, conference abstracts, reviews, and editorials were excluded.

Information Sources

A comprehensive literature search was independently conducted by all authors using multiple electronic databases. The databases searched included PubMed, ScienceDirect, the Cochrane Library, and Google Scholar.

Search Strategy

We used a combination of MeSH terms and free-text keywords. The search string included: (“Magnetic sphincter augmentation” OR “Magnetic sphincter” OR “LINX” OR “magnetic anti-reflux device”) AND (“Laparoscopic fundoplication” OR “Nissen fundoplication” OR “Lap fundoplication”) AND (“Gastroesophageal reflux disease” OR “GERD” OR “acid reflux” OR “heartburn”). The search strategy was applied consistently across all databases. Reference lists of included studies were also screened to identify additional relevant articles.

Study Selection

**Figure 1:**
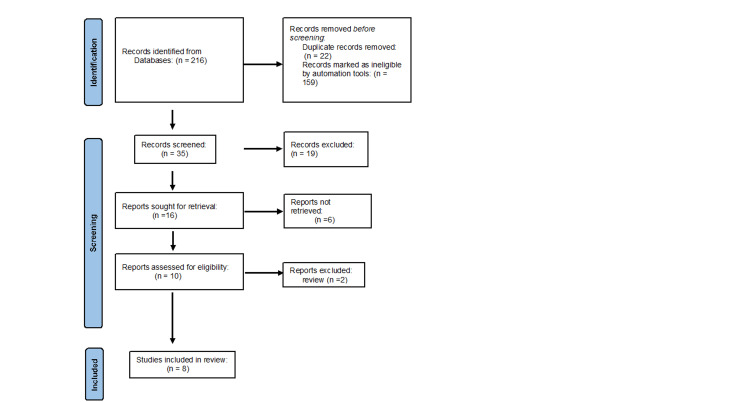
PRISMA Diagram PRISMA: Preferred Reporting Items for Systematic Reviews and Meta-Analyses.

All retrieved articles were screened by two independent reviewers. Titles and abstracts were first reviewed for relevance, followed by full-text screening for eligibility based on predefined inclusion criteria. Any discrepancies were resolved through discussion and, if necessary, consultation with a third senior reviewer. A PRISMA flow diagram (Figure 1) summarizes the study selection process.

Data Extraction

Two reviewers independently extracted data from the included studies using a standardized data extraction form. The variables collected included the first author’s name, year of publication, and country, as well as study design and the number of patients in each intervention group. Patient demographics such as age, body mass index (BMI), hernia size, and esophagitis grade were also recorded. In addition, details regarding the type of surgical procedure (magnetic sphincter augmentation or laparoscopic fundoplication) and the mean operative time (minutes) were extracted. Any discrepancies between the two reviewers were resolved through discussion, and when necessary, a third reviewer was consulted to achieve consensus.

Postoperative outcomes: Gastroesophageal Reflux Disease-Health-Related Quality of Life (GERD-HRQL) scores, dysphagia incidence, proton pump inhibitors (PPIs) discontinuation, ability to belch or vomit, gas/bloating symptoms, patient satisfaction percentage, and complications. Where variables were not reported by study authors, the value was noted as ‘nr’ (not reported) in the tables.

Risk of Bias Assessment

The methodological quality of included studies was assessed using the Newcastle-Ottawa Scale (NOS) for cohort studies [15]. Three reviewers independently scored each study across the three NOS domains: selection, comparability, and outcome. Disagreements were resolved through consensus or adjudication by a senior author.

Data Synthesis

A qualitative synthesis of results was performed due to the heterogeneity of outcome definitions and reporting across studies. Key findings were tabulated, and a narrative summary highlighted comparative outcomes between MSA and LF. Meta-analysis was not performed due to variation in study design and outcome measurement.

Results

Study Selection

A total of eight studies published between 2015 and 2025 met the predefined inclusion criteria. These included adults aged 18 to 65 years with confirmed GERD, non-obese BMI profiles, and comparative cohorts undergoing either magnetic sphincter augmentation (MSA) or laparoscopic fundoplication (LF). All studies were published in English and were accessible without paywalls. Among the eight studies, one was prospective observational study, while the remaining seven were retrospective cohort studies.

Study and Patient Characteristics

Across the eight included studies, a total of 1,598 patients were analyzed, 1,078 (67.4%) underwent MSA, while 520 (32.6%) underwent LF, including Nissen, Toupet, or unspecified approaches. Tables 1, 2 present the demographic, clinical, and procedural characteristics of patients included in the eight comparative studies. All patients underwent surgery through a laparoscopic approach, either magnetic sphincter augmentation (MSA) or fundoplication (Nissen, Toupet, or unspecified LF). The mean age of patients ranged from 39.3 to 61.7 years. The reported mean BMI values ranged from 23.9 to 27.8 kg/m². Mean hiatal hernia size, when reported, ranged from 1.5 to 5.7 cm. Presence of esophagitis Grade B or higher was observed in two to 24 patients per study arm, corresponding to approximately 11.6% (124/1,078) of MSA patients and 12.1% (63/520) of LF patients, where reported. Mean operative time for MSA ranged from 42 to 99.9 minutes, and for LF, it ranged from 65 to 131.3 minutes.

**Table 1:** Demographic Characteristics of Included Studies MSA: magnetic sphincter augmentation; LF: laparoscopic fundoplication; nr: not reported.

Author	Year	Country	Study Design	Type of Surgery	No. of Patients	Mean Age (yrs)	Mean BMI (kg/m²)
Richards and McRae [16]	2018	USA	Observational retrospective	MSA	32	54.5	nr
				Nissen	6	61.7	nr
Riegler et al. [17]	2015	USA	Observational retrospective	MSA	52	53	26
				Nissen	67	53	27
Sheu et al. [18]	2015	USA	Observational retrospective	MSA	12	39.3	26.8
				Nissen	12	43.8	26.8
Asti et al. [19]	2016	Europe	Observational retrospective	MSA	135	44	23.9
				Nissen	103	50	25.1
Reynolds et al. [20]	2016	Europe	Observational retrospective	MSA	202	46.6	25.7
				LF	47	52.8	26.1
Reynolds et al. [21]	2015	USA	Observational retrospective	MSA	50	53	26.4
				Nissen	50	54	26.7
Bonavina et al. [22]	2021	Italy, UK	Observational prospective	MSA	465	46.6	25.7
				LF	166	56.3	27.8
Asti et al. [23]	2023	Italy	Observational retrospective	MSA	130	49.0	25.3
				TF	69	56.6	24.7

**Table 2:** Clinical Characteristics of Included Studies MSA: magnetic sphincter augmentation; LF: laparoscopic fundoplication; nr: not reported.

Author	Year	Type of Surgery	Mean Hernia Size (cm)	Esophagitis ≥ Grade B	Mean OR Time (min)
Richards and McRae [16]	2018	MSA	3.2	nr	90.9
		Nissen	5.7	nr	131.3
Riegler et al. [17]	2015	MSA	nr	2	66
		Nissen	nr	5	82
Sheu et al. [18]	2015	MSA	nr	nr	63.7
		Nissen	nr	nr	90.3
Asti et al. [19]	2016	MSA	2	13	42
		Nissen	2	8	87
Reynolds et al. [20]	2016	MSA	nr	21	nr
		LF	nr	14	nr
Reynolds et al. [21]	2015	MSA	1.5	6	nr
		Nissen	1.6	7	nr
Bonavina et al. [22]	2021	MSA	3.2	2	nr
		LF	5.7	13	nr
Asti et al. [23]	2023	MSA	2	24	65
		TF	2	7	110

Postoperative Outcomes

A narrative synthesis of postoperative outcomes was conducted, as the included studies exhibited heterogeneity in outcome definitions, follow-up duration, and reporting methods. Therefore, meta-analysis was not performed. Key outcome comparisons between magnetic sphincter augmentation (MSA) and laparoscopic fundoplication (LF) are summarized in Table 3. The results reflect variability across studies but demonstrate several consistent trends.

**Table 3:** Postoperative Functional Outcomes and Patient Satisfaction GERD-HRQL: Gastroesophageal Reflux Disease-Health-Related Quality of Life; PPI: proton pump inhibitor; MSA: magnetic sphincter augmentation; LF: laparoscopic fundoplication; nr: not reported.

Type of Surgery	GERD-HRQL	PPI Discontinuation (%)	Dysphagia (n)	Gas Bloating (%)	Ability to Belch (%)	Complications (n)	Patient Satisfaction (%)	
MSA	6.4	nr	nr	nr	93	3	74	
Nissen	8.6	nr	nr	nr	100	0	89	
MSA	3	81.8	30	10	98.4	4	nr	
Nissen	3.5	63	10	31.9	88.9	6	nr	
MSA	nr	nr	83	0	nr	nr	nr	
Nissen	nr	nr	58	33	nr	nr	nr	
MSA	3	nr	nr	nr	nr	nr	nr	
Nissen	3	nr	nr	nr	nr	nr	nr	
MSA	4	41	22	11	90	nr	43	
LF	5	54	30	31	64	nr	54	
MSA	4.2	83	10	27.7	nr	0	nr	
Nissen	4.3	91	13	38.3	nr	2	nr	
MSA	5.2	81	16	nr	96.7	3	75	
LF	4.9	80.3	24	nr	88.5	2	72	
MSA	5	93	3	4	nr	1	nr	
TF	5	80	39	13	nr	1	nr	

GERD-HRQL: Postoperative GERD-HRQL scores improved in both groups, with mean values ranging from 3.0 to 6.4 in MSA patients and 3.0 to 8.6 in LF patients. Preoperative GERD-HRQL scores were statistically comparable across all studies (p > 0.05).

PPI discontinuation: MSA discontinuation rates ranged from 41% to 93%, while LF ranged from 54% to 91%. Of the five studies reporting PPI discontinuation, MSA had numerically higher cessation rates in three [17,22,23], while LF was higher in two [20,21]. Taken together, these data provide a mixed signal and do not demonstrate a consistent advantage for either procedure.

Dysphagia: Persistent dysphagia was more frequently observed in MSA groups, with patient numbers ranging from three to 83. In LF groups, dysphagia incidence ranged from 10 to 30 patients where reported, suggesting a relatively lower frequency than MSA in most studies.

Gas and bloating: Where documented, gas bloating was consistently lower in MSA, with rates between 0% and 27.7%, compared to 31% to 38.3% in LF patients. This suggests MSA provides a functional advantage in preserving physiological gas clearance.

Belching ability: The ability to belch was better preserved in MSA patients, reported as high as 98.4% in one study. LF patients showed lower belching ability, ranging between 64% and 100%, with a trend toward reduced function compared to MSA.

Complications: Postoperative complications were low across all studies. MSA-related complications most commonly including transient dysphagia and chest discomfort, with device erosion, ranged from 0 to 4 cases. A small proportion of patients required device removal due to persistent dysphagia. LF-related complications, including gas-bloat syndrome, dysphagia, and occasional wrap failure, ranged from 0 to 6.

Patient satisfaction: Reported satisfaction ranged from 43% to 75% in MSA patients and 54% to 89% in LF patients. Although satisfaction rates varied, studies focusing on preservation of physiological function tended to favor MSA.

Risk of Bias Assessment

The risk of bias for the included studies was assessed using the Newcastle-Ottawa Scale (NOS) for cohort studies. This tool evaluates the quality of non-randomized studies in three domains: selection (maximum 4 stars), comparability (maximum 2 stars), and outcome (maximum 3 stars), with a maximum possible score of 9. Studies scoring 7-9 were considered high quality, 5-6 as moderate quality, and <5 as low quality. All studies included in this review were judged to have moderate to high methodological quality. The detailed scoring for each study is presented in Table 4.

**Table 4:** Quality Assessment of Included Studies Using the Newcastle-Ottawa Scale (NOS)

Author (Year)	Selection	Comparability	Outcome	Total NOS Score	Comments
Richards and McRae (2018) [16]	4	2	2	8	Well-designed retrospective study but limited by a small Nissen group and brief follow-up.
Riegler et al. (2015) [17]	3	2	2	7	Good comparability, adequate reporting, but outcome assessment less detailed.
Sheu et al. (2015) [18]	3	1	2	6	Small sample size and limited adjustment for confounding.
Asti et al. (2016) [19]	4	2	3	9	Strong design with propensity score matching and comprehensive outcome assessment.
Reynolds et al. (2016) [20]	4	1	2	7	Large sample size but varied centers and patient populations introduce heterogeneity.
Reynolds et al. (2015) [21]	4	2	2	8	Matched-pair design strengthens comparability; moderate sample size.
Bonavina et al. (2020) [22]	4	2	3	9	Large prospective multicenter study with strong methodology and long-term follow-up.
Asti et al. (2023) [23]	4	1	3	8	Good follow-up and outcome detail, but retrospective nature limits comparability.

Discussion

This systematic review synthesized findings from eight comparative studies evaluating the effectiveness and safety of magnetic sphincter augmentation (MSA) versus laparoscopic fundoplication (LF) in the surgical management of gastroesophageal reflux disease (GERD) among non-obese patients. The included studies comprised a total of 1,598 patients and offered a diverse mix of observational designs, including one prospective and seven retrospective studies, with overall moderate to high methodological quality based on the Newcastle-Ottawa Scale.

Both MSA and LF significantly improved GERD-related health outcomes, particularly GERD-HRQL scores, with postoperative improvements consistently noted across studies [16-23]. MSA was associated with specific advantages in preserving physiological functions, especially the ability to belch and reduced gas bloating, benefits emphasized in patient-reported satisfaction outcomes [17,19,22]. These findings are supported by a recent study which concluded that MSA effectively improves GERD across all severity levels, with some outcomes more favorable in mild-to-moderate cases [24]. Another prospective study further supports its efficacy showing favorable outcomes even in patients with >3 cm hernias [25].

PPI discontinuation was numerically higher with MSA in three of five studies [17,22,23] and higher with LF in two [19,20]. This mixed pattern is consistent with prior meta-analytic evidence showing no statistically significant difference in PPI elimination between MSA and LF. A previous systematic review and meta-analysis further supports this, showing no significant difference between MSA and LF in PPI elimination (81.4% vs. 81.5%, p = 0.68) [26]. However, another systematic review and meta-analysis involving 39 studies (n = 8,075) confirmed superior functional outcomes with MSA, including higher rates of PPI discontinuation [27].

Persistent dysphagia was more frequently observed in MSA groups [16,17,19,22], attributed to the fixed ring mechanism of the LINX device. In one study of 380 patients, 15.5% experienced persistent dysphagia at a mean follow-up of 11.5 months, with 31% requiring at least one dilation and 1.8% undergoing device removal. Predictors of dysphagia included preoperative symptoms, weak esophageal motility, and absence of a large hernia, while factors like frequent postoperative eating and larger device size appeared protective [28]. Rarely, persistent dysphagia may result from mechanical failure of the LINX device. One case reported device disruption five years post-implantation, confirmed on imaging, requiring surgical removal and conversion to Toupet fundoplication, which resolved the patient’s symptoms [29].

Gas bloating and inability to belch, common complaints after LF, were consistently reduced in MSA patients [17,19,26]. In a five-year comparative study, both groups showed similar long-term GERD-related quality of life, but MSA patients reported better preservation of belching and gas relief [30]. Patient satisfaction remained high in both groups. However, MSA patients who valued functional preservation (e.g., ability to vomit, absence of bloating) reported higher satisfaction in studies with longer-term follow-up [22,23,28].

The results of this review are aligned with prior reviews and registries indicating that MSA and LF offer similar symptom relief, but MSA may be better tolerated in certain populations particularly non-obese patients, younger individuals, and those prioritizing preservation of physiological functions such as belching and reduced bloating [26,27,30]. Our findings expand on this by emphasizing the experience in non-obese patients, where MSA may serve as a preferable alternative in appropriately selected cases offering reversibility, shorter operative time, and better preservation of functions like belching and vomiting. It may be preferred by younger patients, although careful selection and counseling about early dysphagia are essential. High-resolution manometry is an essential component of the preoperative evaluation for patients being considered for MSA or Nissen fundoplication. It helps rule out major motility disorders, such as achalasia, and characterizes esophageal motility patterns, allowing clinicians to individualize the choice of procedure and reduce the likelihood of postoperative dysphagia. Future high-quality RCTs comparing MSA and LF with standardized outcomes, longer follow-up, and subgroup analysis are needed to further support clinical decisions.

This review focuses specifically on non-obese GERD patients to provide subgroup-level evidence comparing MSA and LF, enabling more tailored clinical interpretation for this population. It adhered to PRISMA guidelines, used dual screening, and applied the Newcastle-Ottawa Scale for quality assessment. However, most studies were retrospective, with limited use of propensity matching and only one prospective study. Heterogeneity in outcome reporting and missing data (marked as “nr”) restricted pooled analysis and required narrative synthesis.

## Conclusions

Both LF and MSA offer effective symptom relief and improved quality of life in non-obese GERD patients, with comparable improvements in GERD-HRQL scores and favorable safety profiles. MSA appears to offer functional advantages such as reduced bloating and preserved ability to belch and vomit, although dysphagia may be more frequent. While MSA shows promise as a surgical alternative, especially for function preservation, further high-quality research is needed to establish its role as a primary procedure in carefully selected patients.
